# Growth Patterns and Factors Associated with Short Stature in Saudi Children and Adolescents with Type 1 Diabetes Mellitus: A Retrospective Cross-Sectional Study

**DOI:** 10.3390/jcm15103629

**Published:** 2026-05-09

**Authors:** Youssef A Alqahtani, Ayed A. Shati, Ayoub Ali Alshaikh, Zeinh Hussein Fardan, Roaa Saad Alhuwail, Dalia Salem A Almosleh, Abeer Mohammed Alshehri, Maram Hadi A Asiri, Shaden Essa Hammati, Reema Abdullah S. Alsharif, Ramy Mohamed Ghazy

**Affiliations:** 1 Department of Child Health, College of Medicine, King Khalid University, Abha 61421, Saudi Arabia; youssef98118911@gmail.com (Y.A.A.); shatiayed@gmail.com (A.A.S.); zali@kku.edu.sa (Z.H.F.); 2 Family and Community Medicine Department, College of Medicine, King Khalid University, Abha 61421, Saudi Arabia; alashaikh@kku.edu.sa; 3 Asser Health Cluster, Ministry of Health, Abha 62523, Saudi Arabia; ro.oa@hotmail.com; 4 College of Medicine, King Khalid University, Abha 61421, Saudi Arabia; daalmosleh@gmail.com (D.S.A.A.); balshhry468@gmail.com (A.M.A.); maram.hadi.asiri@gmail.com (M.H.A.A.); sh.alasire@gmail.com (S.E.H.); alsharifreemay@gmail.com (R.A.S.A.); 5 Health and Medical Research Centre, King Khalid University, Abha 61421, Saudi Arabia; 6 Tropical Health Department, High Institute of Public Health, Alexandria University, Alexandria 21526, Egypt

**Keywords:** type 1 diabetes mellitus, short stature, growth impairment, children and adolescents, diabetes duration, HbA1c, vitamin D, Saudi Arabia

## Abstract

**Background**: The impact of Type 1 Diabetes Mellitus (T1DM) on linear growth in children remains unclear, with conflicting evidence regarding the roles of glycemic control, disease duration, and nutritional factors. This study aimed to assess growth patterns and identify factors independently associated with short stature among Saudi school-age children and adolescents with T1DM, comparing them to a healthy control group. **Methods**: A retrospective cross-sectional study was conducted at an endocrinology clinic, including 250 patients with T1DM aged 5–18 years and 267 healthy controls. After propensity score matching, 231 patients were matched to 231 controls. Data were extracted from electronic medical records using a standardized form. Anthropometric measurements were converted to Z-scores and percentiles using validated Saudi growth charts. Short stature was defined as height below the third percentile for age and gender. Univariate and multivariable logistic regression analyses were performed to identify factors associated with short stature among T1DM participants. **Results**: The median age of the 250 T1DM participants was 13.0 [10.0–15.0] years, with a slight male predominance (58.0%). Of them, 6 (2.4%) children were tall, 30 (12.0% were short), and 214 (85.6%) were normal. A significantly higher proportion of short stature was observed in the T1DM group compared with the control group (6.1% vs. 1.7%; *p* = 0.016). Among T1DM participants, the proportion of short stature increased progressively with diabetes duration: 3.0% in new-onset disease, 14.9% in intermediate duration (2–5 years), and 25.0% in long-standing disease (>5 years) (*p* = 0.001). Age at onset of T1DM was also significantly associated with having short stature (*p* = 0.036). In multivariable analysis, intermediate duration (adjusted odds ratio [aOR] = 5.72, 95% CI (1.02–32.1); *p* = 0.047) and long-standing duration (aOR = 11.03, 95% CI: (1.09–111.45); *p* = 0.042) remained significant independent predictors of short stature. In contrast, glycemic control (HbA1c), vitamin D status, time in range, and treatment adherence were not significantly associated with short stature after adjustment. **Conclusions**: Children and adolescents with T1DM have significantly lower height percentiles and a higher prevalence of short stature compared to their controls. Diabetes duration is a strong, independent predictor of short stature in this population, with progressively higher risk as disease duration lengthens, regardless of glycemic control status. These findings underscore the necessity of systematic longitudinal growth monitoring, particularly in patients with disease duration exceeding five years, to enable early identification and intervention for those at highest risk of growth impairment.

## 1. Introduction

Type 1 diabetes mellitus (T1DM) is the most common type of diabetes diagnosed in children and is often called “childhood-onset” diabetes. T1DM is characterized by insufficient insulin production due to pancreatic beta-cell destruction [1]. The incidence of this disease is dramatically increasing worldwide; the global age-standardized prevalence of T1DM is projected to rise by 23.9%, increasing from 0.2% in 2021 to 0.3% in 2050 [2,3].

T1DM leads to several complications due to poor insulin production and difficulty achieving good blood sugar control. These complications represent a major concern despite the advances in treatment [4]. The main complications are microvascular, including retinopathy, nephropathy, and neuropathy. Patients with T1DM also face diabetic ketoacidosis, especially at diagnosis. In addition, there is an increased risk of cardiovascular disease, higher mortality rates, and a reduced life expectancy compared to people without diabetes [5,6].

Linear growth in children is a complex physiological process influenced by many endocrinological, nutritional, and psychological factors [7,8]. The effects of T1DM on growth are still unclear and debated in the literature [7]. A longitudinal study conducted by Demir et al. [9] found that T1DM did not show a significant overall negative effect on growth; however, growth outcomes were modestly influenced by metabolic control, with poorer control associated with less favorable height changes. On the other hand, some studies showed that T1DM can affect linear growth and pubertal development [4,10,11]. For instance, Mauriac syndrome, characterized by growth failure, hepatomegaly, and poorly controlled diabetes, represents a severe manifestation of impaired growth associated with T1DM [12,13]. Furthermore, evidence indicates that with better glycemic control, normal height development can be maintained [7,14]. Moreover, several studies have reported that children at disease onset are taller than their healthy peers [15], whereas other studies have not observed such differences [16,17]. These discrepancies may be explained by factors such as the secular trend in growth, where children enter puberty earlier than in previous generations, and differences in the selection of control groups [10]. Therefore, monitoring growth in children with T1DM is essential for ensuring optimal long-term health outcomes [18,19].

In Saudi Arabia, there is a scarcity of research assessing the association between stature and T1DM in Saudi Arabia. A retrospective study of 162 pediatric T1DM patients found that long-term hyperglycemia leads to significantly reduced height for age compared to standard cutoffs, with notable gender and age-at-diagnosis variations, particularly affecting females diagnosed at ages 4–10 and males diagnosed at ages 3 or 7–10 years [20]. A cross-sectional study conducted in Jeddah investigated the relationship between metabolic control and growth in children with T1DM. The study found that poor glycemic control (higher HbA1c levels) was associated with lower height, weight, and body mass index (BMI) z-scores [21]. However, to our knowledge, no previous studies have specifically assessed the prevalence of short stature among children with T1DM or evaluated the impact of diabetes duration on growth outcomes. Understanding the determinants of growth impairment in children with T1DM remains limited, particularly in the Middle Eastern context, where data are scarce and existing studies have yielded conflicting results regarding the roles of glycemic control, disease duration, and nutritional factors. To address this gap, we hypothesized that longer diabetes duration and poor glycemic control would be independently associated with short stature, and that modifiable factors, including vitamin D status, treatment adherence, and physical activity, would significantly influence growth outcomes in this population.

## 2. Materials and Methods

### 2.1. Study Design and Setting

This retrospective cross-sectional study was carried out at the endocrinology clinic of King Khalid University Medical City and Abha Maternity and Children Hospital from July 2024 to December 2025.

### 2.2. Sample Size and Study Population

The sample size was calculated to estimate the prevalence of short stature among children with T1DM. Based on a prior study [22], the prevalence of stunting was assumed to be 15.7%. The required sample size was determined using the standard formula for estimating a single proportion:

$$n=\frac{Z^{2}\cdot p(1-p)}{d^{2}}$$

where *n* is the required sample size, *Z* is the standard normal deviate corresponding to a 95% confidence level (1.96), *p* is the estimated prevalence (0.157), and *d* is the desired margin of error (0.05). The minimum calculated sample size was 203 participants. We increased it to 250 to compensate for stratification.

The study population consisted of children and adolescents diagnosed with T1DM, all were on insulin pumps, who attended the endocrinology clinic during the study period. A consecutive sampling method was employed, whereby all eligible patients were included. Patients were included if they were aged between 5 and 18 years, had a confirmed diagnosis of T1DM, had complete anthropometric measurements available, and had accessible clinical and laboratory data. Patients were excluded if they had other chronic illnesses that impact growth, such as genetic syndromic conditions linked to growth disorders, or if their medical records were incomplete. For comparison, we included anthropometric data from 267 healthy children aged 5–18 years. They were recruited from a school-based study conducted in the Aseer region between 2022 and 2023. Control participants were confirmed to be free of chronic illnesses, including diabetes, thyroid disease, growth hormone disorders, and malnutrition, based on review of their medical records and interviews with their parents. Children with a first-degree relative diagnosed with T1DM or any other autoimmune disease were excluded.

### 2.3. Data Collection

Data were obtained from electronic medical records and patient charts using a standardized data collection form. The information collected included:

- Demographic and clinical variables: age, sex, age at diagnosis, duration of diabetes, and family history of diabetes.
- Laboratory measurements: comprised glycated hemoglobin (HbA1c) and vitamin D levels, which were classified as deficient (<20 ng/mL), insufficient (20–29 ng/mL), or sufficient (≥30 ng/mL) based on serum 25-hydroxyvitamin D concentrations according to the Endocrine Society guidelines [23], and time in range defined as the percentage of time blood glucose levels are within the target range (usually 70–180 mg/dL). Based on international consensus for continuous glucose monitoring, time in range was classified as good if ≥70% [24]. Participants with scores below 70% were additionally classified as having fair status (50–69%) or poor (<50%). Lifestyle variables examined comprised balanced diet (Yes/No), adherence to treatment (Yes/No), and physical activity (Yes/No), all based on self-report.
- Anthropometric measurements: data on anthropometric measurements were extracted from medical records in which these data were routinely collected during clinic appointments. All measurements were obtained by trained healthcare staff using calibrated instruments and following standardized procedures. Height was recorded to the nearest 0.1 cm with a wall-mounted stadiometer, with participants standing barefoot, heels together, and their head aligned in the Frankfurt horizontal plane. Weight was recorded to the nearest 0.1 kg using a calibrated digital scale, with participants barefoot and dressed in light clothing. BMI was determined by dividing weight in kilograms by height in meters squared. All values were then expressed as percentiles based on Saudi growth charts matched to participants’ age and sex [25,26].

Definitions and Classifications

Short stature is defined as height below the 3rd percentile for age and gender, normal stature is defined as height between the 3rd and 97th percentiles, and tall stature is defined as height above the 97th percentile. Weight status was categorized as underweight when weight was below the 3rd percentile, normal weight when weight was between the 3rd and 97th percentiles, and overweight when weight was above the 97th percentile.

Glycemic control was categorized based on HbA1c levels, with optimal control defined as HbA1c less than 7.0%, acceptable control as HbA1c between 7.0 and 7.5%, suboptimal control as HbA1c between 7.5 and 9.0%, and poor control as HbA1c greater than 9.0%. Diabetes duration was categorized into new onset for duration of two years or less, intermediate for duration more than two and up to five years, and long-standing for duration greater than five years. Age at diagnosis was categorized into three groups: before 5 years, between 5 and 10 years, and after 10 years.

Statistical Analysis

Statistical analyses were performed using R software (version 4.3.1, Vienna, Austria). All statistical tests were two-tailed, and a *p*-value < 0.05 was considered statistically significant. Descriptive statistics were presented as frequencies and percentages for categorical variables, while continuous variables were presented as median with interquartile range (1QR) for non-normally distributed data. Normality was assessed using the Shapiro–Wilk test. Categorical variables were compared across groups using the chi-square test when assumptions of independence and adequate expected cell counts were met; otherwise, Fisher’s exact test was used. Continuous variables were compared using the Mann–Whitney U test for non-normally distributed data. Comparisons across three or more groups were performed using the Kruskal–Wallis test as appropriate. To minimize confounding by age and sex, we performed 1:1 propensity score matching without replacement between T1DM cases (n = 250) and healthy controls (n = 267). Propensity score matching was derived from a logistic regression model with T1DM status as the outcome and age and sex as predictor variables. We used nearest neighbor matching with exact matching on sex, pairing each T1DM case to the control subject of the same sex whose age was closest. The algorithm iteratively matched each T1DM case to the same-sex control with the smallest absolute age difference. In total, 231 T1DM cases were successfully matched to 231 controls (a 92.4% matching rate). Post-matching covariate balance was evaluated using standardized mean differences for age (SMD < 0.1) and chi-square tests for sex (*p* > 0.05), both indicating satisfactory balance. Univariate logistic regression was performed to identify factors associated with short stature. Variables with a *p*-value less than 0.2 in univariate analysis were included in the multivariate logistic regression model to identify independent predictors of short stature. Second, variables already recognized as clinically important in T1DM research, namely the glycemic control categories (optimal, suboptimal, poor), were retained in the model a priori, irrespective of their univariable *p*-values, to minimize confounding by indication and maintain clinical interpretability [27]. Results were presented as odds ratios with 95% confidence intervals. Model fit was assessed using the Hosmer–Lemeshow goodness-of-fit test, predictive performance was evaluated using the area under the receiver (AUC) operating characteristic curve, and McFadden’s pseudo-R-squared was calculated to assess model explanatory power.

Ethical Considerations

This study was conducted in accordance with the Declaration of Helsinki. To ensure confidentiality and privacy, all patient data were de-identified prior to analysis. The Institutional Review Board of King Khalid University reviewed and approved the study protocol (IRB No. KKU-175-2025-31). Approved on 18 December 2025. Given the retrospective nature of the study and the use of de-identified medical records, the Institutional Review Board waived the requirement for informed consent.

## 3. Results

A total of 250 children with T1DM were included in this study, with a median age of 13.0 [10.0–15.0] years. The study population consisted predominantly of adolescents aged 10–14 years (42.8%), with a slight male predominance (58.0%). Approximately three-fifths of participants had an intermediate duration of diabetes (65.2%), with a median duration of 2.0 years (IQR: 1.0–4.0) in the overall sample. Glycemic control was generally poor, with 147 (58.8%) participants classified as poorly controlled and a median HbA1c of 9.6% (IQR: 7.7–11.5) in the sample. Vitamin D insufficiency and deficiency were common, affecting approximately two-thirds of the cohort. The median serum level in the total sample was 26.0 ng/mL (IQR: 19.0–36.0). While most participants reported adherence to treatment (81.6%) and a balanced diet (86.8%), the majority were categorized as physically inactive (82.8%), Table 1.

After performing propensity score matching, 231 children were included in each group (control and T1DM). Table 2 demonstrates significant differences between the matched control and T1DM groups across most anthropometric parameters. Height category showed a significant association with a higher proportion of short stature in T1DM (6.1% vs. 1.7%) (*p* = 0.016, Cramér’s V = 0.11). The T1DM group had a higher proportion of underweight individuals (3.0% vs. 0%) (*p* = 0.001, Cramér’s V = 0.153). BMI categories were significantly different across groups (*p* = 0.024, Cramér’s V = 0.142). For continuous variables, the T1DM group had significantly lower height percentiles (median 49.3 vs. 84.5, *p* < 0.001, r = 0.35) and weight percentiles (58.2 vs. 78.6, *p* < 0.001, r = 0.25). In contrast, BMI percentiles did not differ significantly between groups (62.5 vs. 74.2, *p* = 0.101, r = 0.08). Absolute height was slightly lower in the T1DM group (148.0 vs. 151.3 cm, *p* = 0.023, r = 0.11), while weight (44.0 vs. 46.4 kg, *p* = 0.140) and BMI (19.0 vs. 20.5 kg/m^2^, *p* = 0.353) showed no statistically significant differences.

Age-stratified analysis revealed that height differences were not significant in the 5–9 years group (*p* = 0.217) but became significant in the 10–14 years group (*p* < 0.001, r = 0.32) and more pronounced in the 15–18 years group (*p* < 0.001, r = 0.64). A similar pattern was observed for height percentiles, with the largest difference in the 15–18 years group (45.9 vs. 97.1, *p* < 0.001, r = 0.73). Weight was significantly lower in the T1DM group beginning in the 5–9 years group (*p* = 0.012, r = 0.27) and remained lower across older age groups. Weight percentiles in the T1DM group were consistently lower across all age categories compared to controls. BMI showed variable patterns across age groups. In the 5–9 years group, BMI was slightly higher in the T1DM group (*p* = 0.018, r = −0.25), whereas in older groups, BMI tended to be higher in controls, reaching statistical significance in the 15–18 years group (*p* = 0.004, r = 0.26).

Anthropometric measurements demonstrated a clear age-related increase in absolute height, weight, and BMI across all age groups. Median height increased from 119.0 cm [111.0–126.0] in the 5–9 years group to 144.0 cm [136.0–152.0] in the 10–14 years group and 157.0 cm [150.0–167.0] in the 15–18 years group (*p* < 0.001). A similar progressive increase was observed for weight, rising from 20.0 kg [18.0–25.0] to 40.0 kg [31.0–50.0] and 57.0 kg [47.5–65.0] across the respective age groups (*p* < 0.001). BMI also increased significantly with age, from 18.0 kg/m^2^ [14.2–18.0] in younger children to 18.6 kg/m^2^ [16.0–21.9] and 21.9 kg/m^2^ [19.0–25.9] in adolescents (*p* < 0.001). In contrast, anthropometric percentile measures showed no statistically significant differences across age groups. Height percentile demonstrated a non-significant decreasing trend from 54.9 [25.6–84.5] in the 5–9 years group to 46.2 [8.5–75.8] and 44.6 [10.3–71.4] in older age groups (*p* = 0.13). Similarly, weight percentile remained relatively stable across age groups (*p* = 0.9), and BMI percentile showed a non-significant decline from 78.4 [43.4–93.1] to 61.0 [31.2–78.7] and 57.3 [32.5–83.6] (*p* = 0.086), Table 3.

Of the 250, 6 (2.4%) children were tall (excluded), 30 (12.0% were short), and 214 (85.6%) were normal. Diabetes duration was significantly associated with stature status (*p* = 0.004), with the proportion of short stature increasing progressively from new-onset diabetes (3.0%) to intermediate duration (14.9%) and long-standing diabetes (25.0%). Age at onset of T1DM showed a statistically significant association with stature status (*p* = 0.036), with the highest proportion of short stature observed among children diagnosed between 5 and 10 years of age (17.5%), compared to those diagnosed before 5 years (12.5%) and after 10 years (6.5%). Vitamin D status showed a significant association with stature status (*p* = 0.042), with a higher proportion of short stature among participants with vitamin D insufficiency (19.3%), compared to deficiency (11.0%) and sufficiency (6.8%). However, median vitamin D levels were comparable between groups (*p* = 0.447). Age group was not significantly associated with stature status (*p* = 0.126), although the highest proportion of short stature was observed in the 10–14 years group (17.5%) compared to the 5–9 years (8.2%) and 15–18 years (8.7%) groups. No significant association was observed between glycemic control and stature status (*p* = 0.534), although short stature was more frequent in the suboptimal (17.0%) and poor control groups (13.9%) compared to the optimal group (6.9%). HbA1c levels were similar between groups (*p* = 0.173). Balanced diet (*p* = 0.144), time in range (*p* = 0.19), treatment adherence (*p* = 0.358), gender (*p* = 0.705), and family history of diabetes (*p* = 1.0) were not significantly associated with stature status Table 4.

Compared with new-onset disease, the odds of short stature were significantly higher among individuals with intermediate diabetes duration in both unadjusted (OR = 5.69, 95% CI: 1.31–24.82; *p* = 0.021) and adjusted analyses (aOR = 5.72, 95% CI: 1.02–32.10; *p* = 0.047). The association was stronger in long-standing diabetes, remaining significant in both unadjusted (OR = 10.83, 95% CI: 1.78–65.91; *p* = 0.010) and adjusted models (aOR = 11.03, 95% CI: 1.09–111.45; *p* = 0.042). Age at onset of T1DM was not significantly associated with short stature after adjustment. Although onset after 10 years was associated with lower odds in the unadjusted analysis (OR = 0.33, 95% CI: 0.13–0.80; *p* = 0.015), this association was not sustained in the adjusted model (aOR = 0.50, 95% CI: 0.13–1.94; *p* = 0.316). Before five years showed no significant association in either model. Vitamin D status was also not independently associated with short stature. Although insufficiency showed higher odds in the unadjusted model (OR = 3.26, 95% CI: 1.21–8.80; *p* = 0.019), this effect was attenuated after adjustment (aOR = 1.51, 95% CI: 0.43–5.36; *p* = 0.524), while deficiency showed no significant association, Table 5. The Akaike Information Criterion (AIC) was 179.54, and the Bayesian Information Criterion (BIC) was 228.50. The final model achieved a log-likelihood of −75.77, compared with a null deviance of 181.91 and a residual deviance of 151.54, indicating improved model fit after including the predictors. The model exhibited moderate explanatory capacity, with a McFadden R^2^ of 0.167, a Cox and Snell R^2^ of 0.117, and a Nagelkerke R^2^ of 0.223. The likelihood ratio test showed that the model was statistically significant overall (*p* < 0.05). In addition, the Hosmer–Lemeshow goodness-of-fit test was non-significant (χ^2^ = 6.92, df = 8, *p* = 0.546).

**Figure 1:**
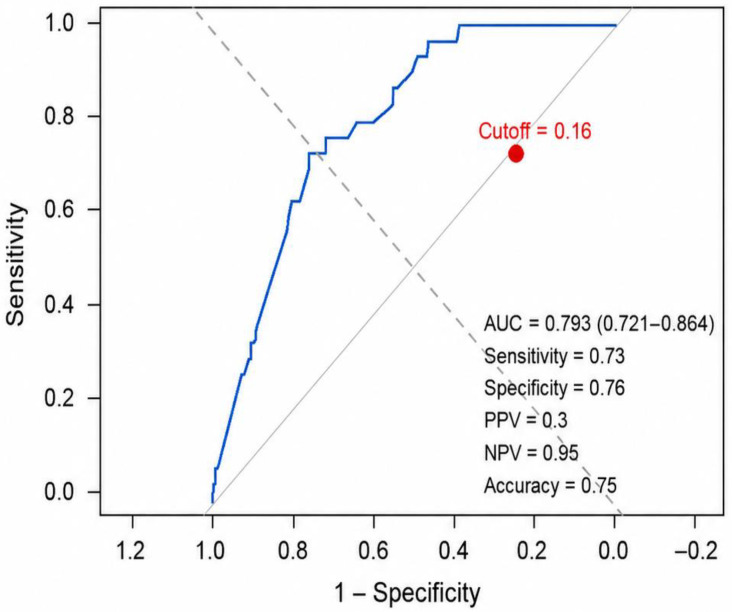
Roc curve for multivariate logistic regression model predicting short stature in children with T1DM. Solid blue line: Receiver Operating Characteristic (ROC) curve showing the trade-off between sensitivity and 1 − specificity across all possible cutoff values. The closer the curve is to the upper-left corner, the better the diagnostic performance. Gray solid line: Line of no discrimination (random classifier): represents a classifier with no predictive ability (AUC = 0.5). Dashed gray line (Optimal cutoff boundary): a line perpendicular to the line of no discrimination used to identify the point on the ROC curve that is farthest from random classification. Red point: Selected cutoff value (0.16) on the ROC curve, with its corresponding sensitivity and specificity. Performance measures: AUC, sensitivity, specificity, positive predictive value (PPV), negative predictive value (NPV), and accuracy at the selected cutoff.

The multivariable logistic regression model demonstrated moderate discriminative ability in identifying short stature among patients with T1DM, with an AUC of 0.793 (95% CI: 0.721–0.864). At the optimal probability threshold of 0.16, the model achieved a sensitivity of 73.0% and a specificity of 76.0%, with an overall accuracy of 75.0%. The negative predictive value (NPV) was high at 95%, indicating strong performance in correctly ruling out short stature among individuals with predicted probabilities below the cut-off. In contrast, the positive predictive value (PPV) was relatively low at 29.7%, likely reflecting the low prevalence of short stature in the study population, Figure 1.

## 4. Discussion

This retrospective cross-sectional study of 250 children and adolescents with T1DM found that while most participants had normal anthropometric measurements, 12.0% were classified with short stature. The key factor associated with short stature was diabetes duration, with the proportion of short stature increasing progressively with longer disease duration. Multivariable logistic regression confirmed that intermediate and long-standing diabetes duration were significant independent predictors of short stature. In contrast, HbA1c and vitamin D status were not significantly associated with stature status in the adjusted analysis.

Prevalence of growth delay among children with T1DM: In the current study, the prevalence of short stature among children with T1DM was 12.0%. A similar prevalence of moderate (11.3%) and severe short (1.8%) stature was reported by El Mozun et al. [25] in a large community-based study. A similar prevalence of 15.7% was reported in India among children aged 1–18 years [22]. On the other hand, a study in Iraq by Qadir et al. [28] reported a higher prevalence of stunting at 33.8%. Also, a study in Mexico found that 50% of children with T1DM were stunted [11]. The observed differences in the prevalence of growth delay across the studies may stem from the wide variation in the sample size and the different tools and methods used to measure height. The growth delay among children with T1DM may be attributed to multiple factors including insufficient insulin therapy, the presence of coexisting autoimmune conditions such as thyroiditis, celiac disease, Addison’s disease, pernicious anemia, hypoparathyroidism, hypergonadotropic hypogonadism, alopecia, vitiligo, and renal function, which can disrupt metabolic and hormonal balance and psychosocial factors, which may indirectly affect growth through effects on nutrition, stress, and overall well-being [29].

Interestingly, after applying propensity score matching, we observed a significant difference in height-related measures between children and adolescents with T1DM and healthy controls. The strong association with the height category with increasing age and the gradual decline in height percentiles among participants with T1DM suggest that the disease duration may have influenced linear growth. Notably, height differences were not apparent in younger children but became more evident with increasing age. Together with the larger effect sizes seen in older age groups, this pattern suggests that growth impairment in T1DM is cumulative and becomes more apparent with longer disease duration. This increasing effect size underscores the clinical importance of this finding. Moreover, in multivariable analysis, the study duration of T1DM was the most important factor that negatively affected the height of affected children. Similar findings were reported in the literature [11]. It has been shown that, particularly during puberty, patients with T1DM exhibit disturbances in the growth hormone (GH)/insulin-like growth factor-1 (IGF-1) axis. These changes include increased GH secretion, decreased serum levels of IGF-1 and IGFBP-3, and elevated IGFBP-1 concentrations [30]. The reduction in IGF-1 levels is thought to result from low portal insulin concentrations, as subcutaneous insulin therapy cannot fully replicate physiological pancreatic insulin delivery to the portal circulation [31]. Several studies have reported that the lowest IGF-1 levels were observed in children with the highest HbA1c levels [32,33]. In addition, other research has shown that circulating IGF-1 levels increase following improved glycemic control achieved through intensive insulin therapy [34,35].

In this study, although body weight was consistently lower in the T1DM group across all age categories, BMI did not exhibit a consistent pattern. The comparatively higher BMI found in younger children with T1DM, followed by lower BMI in older age groups relative to controls, indicates that BMI is influenced by multiple determinants, such as growth velocity, pubertal development, and treatment-related factors. The reduced prevalence of overweight and obesity in the T1DM group, despite comparable or slightly elevated BMI in certain subgroups, suggests that BMI classification may be capturing reduced height rather than increased adiposity [36]. This is particularly critical when interpreting BMI in pediatric patients with chronic diseases that impair normal growth.

In this study, we found that the prevalence of short stature was higher among children diagnosed before the age of 10 years compared to older and younger age groups. However, this significant association did not remain in multivariate analysis. On the other hand, Brown et al. [10] reported that children diagnosed with T1DM between the ages of five and ten tend to be taller than healthy controls; however, no significant differences were observed in cases with earlier or later disease onset. Similarly, a large-scale study by Bonfing et al. [37] found that the mean height standard deviation score (SDS) at diagnosis was significantly higher than that of the general healthy population. These findings have led researchers to investigate whether early growth patterns may be a risk factor for T1DM.

In this study, the prevalence of short stature increased with worsening glycemic control; however, this association was not statistically significant. It may be explained by several methodological and physiological considerations. To begin with, HbA1c reflects average blood glucose over only the previous 2–3 months, whereas linear growth is a long-term, cumulative process shaped by metabolic conditions over years rather than weeks [7]. As diabetes duration emerged as the strongest predictor of short stature, it likely serves as a proxy for cumulative glycemic exposure (collinearity between the two variables), which a single cross-sectional HbA1c measurement cannot capture. Second, the GH/IGF-1 axis disturbances characteristic of T1DM, including increased GH secretion and decreased IGF-1 levels, may persist even in the setting of acceptable HbA1c values, as subcutaneous insulin therapy cannot fully replicate physiological portal insulin delivery to the liver [31]. IGF-1 levels correlate more strongly with diabetes duration and cumulative metabolic control than with isolated HbA1c measurements [38]. Third, the study population exhibited uniformly poor glycemic control overall (median HbA1c 9.6%, with 58.8% classified as poorly controlled), resulting in limited variability that may have reduced the ability to detect a significant association. Finally, as noted by Santi et al. [7], with contemporary diabetes management and intensive insulin regimens, many children with T1DM achieve normal growth regardless of moderate variations in HbA1c, suggesting that factors such as disease duration, timing of onset, and nutritional status may exert more direct effects on linear growth than short-term glycemic metrics alone.

The finding of no significant association between vitamin D status and short stature should be interpreted with caution. Serum 25(OH)D was assessed only once, even though vitamin D levels are known to fluctuate seasonally, typically reaching their lowest values in winter because of decreased sunlight exposure in the Aseer region [39]. The lack of adjustment for the season of blood sampling or for patterns of sun exposure may have led to non-differential misclassification, which could have diluted a genuine association. Furthermore, the significant p-value for vitamin D insufficiency in the univariate analysis (*p* = 0.019), together with the wide confidence interval around the adjusted odds ratio (aOR = 1.51, 95% CI: 0.43–5.36), points to substantial statistical imprecision, most likely driven by small subgroup numbers rather than a true absence of biological effect.

Implication of the study: Based on the findings of this study, several important implications emerge for clinical practice and patient management. First, the strong independent association between longer diabetes duration and short stature underscores the need for systematic and longitudinal growth monitoring, particularly in children who have lived with T1DM for more than five years, as this group faces the highest risk of growth impairment. Second, the absence of a significant association between HbA1c levels and short stature suggests that glycemic control alone is an insufficient predictor of growth outcomes; clinicians should not rely solely on HbA1c as a proxy for normal linear development. Finally, these findings support the need for proactive, multidisciplinary approaches that incorporate regular growth assessments, nutritional optimization, and physical activity promotion to mitigate the cumulative impact of chronic disease on childhood development.

Strengths and limitations: This multicenter study demonstrates several important strengths, including the application of a standardized data collection form, the use of validated Saudi growth charts for anthropometric categorization, and a rigorous statistical framework that employed propensity score matching, univariate and multivariable logistic regression with appropriate evaluations of model fit. The detailed inclusion of clinical, laboratory, and lifestyle parameters such as HbA1c, vitamin D levels, time in range, and self-reported adherence enabled a multidimensional assessment of factors linked to short stature. Finally, we also acknowledge that although the study design restricts causal interpretation, an important strength is the inclusion of an age- and sex-matched healthy control group. This comparison clearly shows that children with T1DM have markedly lower height percentiles, a finding that stands on its own and does not depend on causal inference. However, several limitations must be acknowledged. The retrospective cross-sectional design precludes causal inference and examination of growth trajectories over time. Our cross-sectional measurement of height at a single time precluded assessment of growth velocity, and we lacked data on parental height (to determine familial short stature), pre-diagnosis growth records, pubertal staging (Tanner), bone age, and bone mineral density, all essential for comprehensive growth evaluation. We did not systematically screen for autoimmune comorbidities (celiac disease, hypothyroidism) or assess markers of malabsorption and nutritional deficiencies. Regarding glycemic control, a single HbA1c measurement reflects only 2–3 months and shows limited variability; we lacked average HbA1c over 12–24 months, as well as continuous glucose-monitoring-derived metrics, including coefficient of variation (CV), time below range (TBR), and time above range (TAR). Lifestyle factors (diet, physical activity, adherence) were assessed using simple yes/no responses from medical records, introducing recall and social desirability bias. No quantitative dietary intake (energy, protein) was assessed, and socioeconomic status and parental education were not collected. All participants used insulin pumps, precluding comparison with multiple daily injection therapy. Insulin dose (units/kg/day) was not available. Finally, small subgroup sizes (e.g., long-standing diabetes, n = 8; short stature, n = 30) resulted in wide confidence intervals and limited statistical power. Accordingly, our findings should be viewed as hypothesis-generating rather than confirmatory.

## 5. Conclusions

This study demonstrates that 12% of children with T1DM were short. Diabetes duration is a significant independent predictor of short stature in children and adolescents with T1DM, with the prevalence of growth impairment increasing progressively in new-onset cases compared to those with long-standing disease. Notably, while poor glycemic control and vitamin D insufficiency were common in the cohort, neither emerged as a statistically significant predictor of short stature in the multivariable analysis. Clinically, these results underscore the importance of longitudinal growth monitoring throughout the course of T1DM, particularly beyond the first five years following diagnosis, to enable early identification and intervention for at-risk children. Future prospective studies are warranted to elucidate the mechanistic pathways linking diabetes duration to growth failure.

## Figures and Tables

**Table 1:** Baseline demographic and clinical characteristics of the study population (n = 250).

Variable	Category	T1DM (n = 250)
Age group (years)	5–9	51 (20.4%)
	10–14	107 (42.8%)
	15–18	92 (36.8%)
	Median [Q1–Q3]	13.0 [10.0–15.0]
Gender	Male	145 (58.0%)
	Female	105 (42.0%)
Diabetes duration (clinical category)	New onset	71 (28.4%)
	Intermediate	163 (65.2%)
	Long-standing	16 (6.4%)
	Median [Q1–Q3]	2.0 [1.0–4.0]
Age at diagnosis	Before 5 years	16 (6.4%)
	5–10 years	126 (50.4%)
	After10 years	108 (43.2%)
	Median [Q1–Q3]	10.0 [7.0–12.0]
Glycemic control	Optimal	29 (11.6%)
	Acceptable	24 (9.6%)
	Suboptimal	50 (20.0%)
	Poor	147(58.8%)
HbA1c (%)	Median [Q1–Q3]	9.6 [7.7–11.5]
Serum vitamin D concentration	Deficient	75 (30.0%)
	Insufficient	85 (34.0%)
	Sufficient	90 (36.0%)
	Median [Q1–Q3]	26.0 [19.0–36.0]
Balanced diet (yes)		217 (86.8%)
Treatment adherence (yes)		204 (81.6%)
Family history of diabetes (yes)		54 (21.6%)
Physical activity (yes)		43 (17.2%)

**Table 2:** Comparison of anthropometric measurements between normal and T1DM groups after propensity score matching (231 normal vs. 231 T1DM).

Domain/Age Group	Variable/Category	T1DM (n = 231)	Control (n = 231)	Test Statistic	*p*	Effect Size (r)
Overall	Height Category Normal/tall	217 (93.9%)	227 (98.3%)	χ^2^ = 5.79	<0.016	0.11
	Short stature	14 (6.1%)	4 (1.7%)			
	Height Percentile	49.3 [20.9–77.2]	84.5 [42.5–97.1]	W = 37,364	<0.001	0.35
	Height (cm)	148.0 [133.0–156.5]	151.3 [130.6–166.6]	W = 29,937	0.023	0.11
	Weight Status–Normal	215 (93.1%)	209 (90.5%)	χ^2^ = 10.82	0.001	0.153
	Underweight	7 (3.0%)	0 (0%)			
	Overweight	9 (3.9%)	18 (7.8%)			
	Weight Percentile	58.2 [34.2–79.5]	78.6 [50.8–92.3]	W = 34,347	<0.001	0.25
	Weight (kg)	44.0 [29.0–56.5]	46.4 [31.3–61.0]	W = 28,796	0.14	0.07
	BMI Classification–Normal	171 (74.0%)	142 (61.5%)	χ^2^ = 9.34	0.025	0.142
	Thinness	9 (3.9%)	18 (7.8%)			
	Overweight	33 (14.3%)	42 (18.2%)			
	Obese	18 (7.8%)	29 (12.6%)			
	BMI Percentile	62.5 [33.1–83.2]	74.2 [33.0–88.8]	W = 29,036.5	0.101	0.08
	BMI (kg/m^2^)	19.0 [17.0–22.6]	20.5 [16.6–23.6]	W = 28,012	0.353	0.04
5–9 years	Height (cm)	119 [112–125.2]	122 [111.7–130.8]	U = 1561.5	0.217	0.13
	Weight (kg)	20 [18–24]	25.2 [18.8–31.6]	U = 1316.5	0.012	0.27
	BMI (kg/m^2^)	18.5 [16–23]	18.2 [13.4–21]	U = 2258	0.018	−0.25
	Height Percentile	55.5 [26.9–82.9]	52.1 [18.4–93.3]	U = 1813.5	0.946	−0.01
	Weight Percentile	46.1 [37–77.8]	70.6 [26.3–95.2]	U = 1544	0.185	0.14
10–14 years	Height (cm)	145.0 [138–152]	151.7 [144.6–158.7]	U = 2529	<0.001	0.32
	Weight (kg)	42.0 [32.4–50]	47.3 [38.7–54.7]	U = 2755	0.004	0.26
	BMI (kg/m^2^)	19 [17.1–22.2]	21.0 [17.0–23.8]	U = 3130.5	0.08	0.16
	Height Percentile	50.9 [19.2–76.7]	85.1 [37.0–97.0]	U = 2240.5	<0.001	0.4
	Weight Percentile	59.2 [32.4–82.4]	79.9 [57.3–91.8]	U = 2528.5	<0.001	0.32
15–18 years	Height (cm)	158.5 [151–167.2]	175.5 [164.7–184.8]	U = 1221.5	<0.001	0.64
	Weight (kg)	57.0 [48.0–65.0]	68.1 [53.9–79.0]	U = 2169.5	<0.001	0.37
	BMI (kg/m^2^)	19.0 [17.0–24.0]	22.4 [19.0–25.6]	U = 2532.5	0.004	0.26
	Height Percentile	45.9 [14.4–72]	97.1 [72.8–97.2]	U = 922.5	<0.001	0.73
	Weight Percentile	59.2 [31.6–78.1]	80.8 [59.5–92.4]	U = 2117.5	<0.001	0.38

Chi-square (χ^2^) tests were used to examine associations between categorical variables (height category, weight status, and BMI category) across groups. Effect size for chi-square analyses was reported using Cramér’s V. Cramér’s V values were interpreted as small (~0.10), medium (~0.30), and large (~0.50). Thinness is defined as a BMI-for-age below the 5th percentile, normal weight as the 5th to <85th percentile, overweight as the 85th to <95th percentile, and obesity as ≥95th percentile. Continuous variables (height, weight, and BMI percentiles) were compared between groups using the Mann–Whitney U test due to non-normal distribution, with effect sizes reported as rank-biserial correlation (r). A *p*-value < 0.05 was considered statistically significant.

**Table 3:** Anthropometric characteristics of age group and gender (n = 250).

Characteristic	Category	Overall [n = 250]	5–9 Years [n = 51]	10–14 Years [n = 107]	15–18 Years [n = 92]	*p*-Value
Gender	Male	145 (58.0%)	27 (52.9%)	60 (56.1%)	58 (63.0%)	0.40
	Female	105 (42.0%)	24 (47.1)	47 (43.9%)	34 (37.0%)	
Height [cm]	Median [Q1–Q3]	146.0 [132.0–156.0]	119.0 [111.0–126.0]	144.0 [136.0–152.0]	157.0 [150.0–167.0]	<0.001
Height Percentile	Median [Q1–Q3]	47.3 [14.3–76.5]	54.9 [25.6–84.5]	46.2 [8.5–75.8]	44.6 [10.3–71.4]	0.13
Weight [kg]	Median [Q1–Q3]	42.5 [28.0–55.0]	20.0 [18.0–25.0]	40.0 [31.0–50.0]	57.0 [47.5–65.0]	<0.001
Weight Percentile	Median [Q1–Q3]	56.6 [31.1–79.5]	48.4 [35.0–78.0]	56.4 [25.0–82.1]	57.5 [31.1–77.2]	0.9
BMI [kg/m^2^]	Median [Q1–Q3]	19.0 [17.0–22.5]	18.0 [14.2–18.0]	18.6 [16.0–21.9]	21.9 [19.0–25.9]	<0.001
BMI Percentile	Median [Q1–Q3]	62.1 [32.6–85.2]	78.4 [43.4–93.1]	61.0 [31.2–78.7]	57.3 [32.5–83.6]	0.086

BMI, body mass index; cm, centimeters; kg, kilograms; kg/m^2^, kilograms per meter squared; Q1, first quartile (25th percentile); Q3, third quartile (75th percentile). *p*-values were calculated using the Kruskal–Wallis test for continuous variables (height, weight, BMI, and all percentiles) and the chi-square test for gender.

**Table 4:** Comparison of sociodemographic and clinical characteristics by stature status (normal vs. short stature) (n = 244).

Variable	Category	Normal Stature (n = 214)	Short Stature (n = 30)	Test Statistics, Effect Size, *p*-Value
Age Group (years)	5–9	45 (91.8%)	4 (8.2%)	χ^2^ = 4.44, V = 0.135, *p* = 0.126
	10–14	85 (82.5%)	18 (17.5%)	
	15–18	84 (91.3%)	8 (8.7%)	
Gender	Male	126 (88.7%)	16 (11.3%)	χ^2^ = 0.14, φ = 0.024, *p* = 0.705
	Female	88 (86.3%)	14 (13.7%)	
Diabetes Duration	New onset	65 (97.0%)	2 (3.0%)	χ^2^ = 8.8, V = 0.19, *p* = 0.004
	Intermediate	137 (85.1%)	24 (14.9%)	
	Long-standing	12 (75.0%)	4 (25.0%)	
Glycemic Control	Optimal	27 (93.1%)	2 (6.9%)	χ^2^ = 5.46, V = 0.145, *p* = 0.141
	Acceptable	24 (100.0%)	0 (0.0%)	
	Suboptimal	39 (83.0%)	8 (17.0%)	
	Poor	124 (86.1%)	20 (13.9%)	
HbA1c (%)	Median [Q1–Q3]	9.6 [7.5–11.5]	9.6 [8.4–12.4]	W = 2716.5, r = 0.09, *p =* 0.173
Age at Onset of T1DM	Before 5 years	14 (87.5%)	2 (12.5%)	χ^2^ = 6.4, V = 0.162, *p* = 0.036
	5–10 years	99 (82.5%)	21 (17.5%)	
	After 10 years	101 (93.5%)	7 (6.5%)	
Serum vitamin D concentration	Deficient	65 (89.0%)	8 (11.0%)	χ^2^ = 6.32, V = 0.161, *p* = 0.042
	Insufficient	67 (80.7%)	16 (19.3%)	
	Sufficient	82 (93.2%)	6 (6.8%)	
	(ng/mL) Median [Q1–Q3]	26 [19–37]	25.5 [19.2–28]	W = 3485.5, r = 0.05, *p* = 0.447
Balanced Diet	Yes	183 (86.3%)	29 (13.7%)	χ^2^ = 1.98, φ = 0.09, *p* = 0.144
	No	31 (96.9%)	1 (3.1%)	
Time in Range (TIR)	Poor	121 (84.6%)	22 (15.4%)	χ^2^ = 3.32, V = 0.117, *p* = 0.19
	Fair	36 (90.0%)	4 (10.0%)	
	Good	57 (93.4%)	4 (6.6%)	
Treatment Adherence	Yes	176 (88.9%)	22 (11.1%)	χ^2^ = 0.85, φ = 0.059, *p* = 0.358
	No	38 (82.6%)	8 (17.4%)	
Family History of Diabetes	Yes	46 (86.8%)	7 (13.2%)	χ^2^ = 0, φ = 0, *p* = 1
	No	168 (88.0%)	23 (12.0%)	

Total N = 244 after excluding patients with tall stature (height > 97th percentile) from the original 250 participants; HbA1c, glycated hemoglobin; Q1, first quartile (25th percentile); Q3, third quartile (75th percentile).

**Table 5:** Factors associated with short stature among study participants: univariable and multivariable logistic regression analysis.

Variable	Category	Univariable OR (95% CI)	*p*	Adjusted OR (95% CI)	*p*
Age group	5–9 years	Reference		Reference	
	10–14 years	2.38 (0.76–7.46)	0.136	1.74 (0.38–7.92)	0.472
	15–18 years	1.07 (0.31–3.75)	0.914	1.34 (0.17–10.25)	0.781
Diabetes duration	New onset	Reference		Reference	
	Intermediate	5.69 (1.31–24.82)	0.021	5.72 (1.02–32.1)	0.047
	Long standing	10.83 (1.78–65.91)	0.01	11.03 (1.09–111.45) ^@^	0.042
Glycemic control	Good ^#^	Reference		Reference	
	Suboptimal	5.23 (1.05–26.02)	0.043	3.79 (0.39–36.54)	0.25
	Poor	4.11 (0.93–18.23)	0.063	0.78 (0.03–17.55)	0.875
Age at onset of T1DM	5–10 years	Reference		Reference	
	Before 5 years	0.67 (0.14–3.19)	0.618	0.90 (0.12–6.64)	0.917
	After 10 years	0.33 (0.13–0.8)	0.015	0.51 (0.13–1.94)	0.316
Time in range	Good	Reference		Reference	
	Fair	1.58 (0.37–6.73)	0.534	0.62 (0.09–4.14)	0.608
	Poor	2.59 (0.85–7.87)	0.093	4.92 (0.36–66.48)	0.23
Serum vitamin D concentration	Sufficient	Reference		Reference	
	Insufficient	3.26 (1.21–8.8)	0.019	1.51 (0.43–5.36)	0.524
	Deficient	1.68 (0.56–5.09)	0.357	0.81 (0.2–3.21)	0.761
Balanced Diet	Yes	Reference		Reference	
	No	0.23 (0.03–1.55)	0.124	0.24 (0.03–2.03)	0.191

# acceptable and optimal control were merged into good, @: The wide confidence intervals observed in the multivariable analysis may reflect limited sample size and should be interpreted with caution. Abbreviations: CI, confidence interval; OR: odds ratio; T1DM, type 1 diabetes mellitus. Multivariable model included variables with *p* < 0.2 in univariable analysis and glycemic control.

## Data Availability

Data is available upon request by contacting the corresponding author.

## Abbreviations

**Abbreviation**	**Full Form**
aOR	Adjusted odds ratio
BMI	Body mass index
CI	Confidence interval
cm	Centimeter
GH	Growth hormone
HbA1c	Glycated hemoglobin
IGF-1	Insulin-like growth factor-1
IGFBP-1	Insulin-like growth factor-binding protein 1
IGFBP-3	Insulin-like growth factor-binding protein 3
IQR	Interquartile range
IRB	Institutional Review Board
ISPAD	International Society for Pediatric and Adolescent Diabetes
kg	Kilogram
kg/m^2^	Kilograms per meter squared
ng/mL	Nanograms per milliliter
OR	Odds ratio
Q1	First quartile (25th percentile)
Q3	Third quartile (75th percentile)
SD	Standard deviation
SDS	Standard deviation score
T1DM	Type 1 diabetes mellitus
TIR	Time in range
